# South Africa–Cuba medical training programme: flawed but successful

**Published:** 2018

After more than 20 years, the contentious South Africa– Cuba medical training programme is being downscaled. Business Day’s Tamar Kahn assessed the situation as the largest-ever cohort of 720 students prepares to return to embark on local clinical training.

More than 20 years ago, Desmond Kegakilwe left Tlakgameng village in rural North West to study medicine in Cuba. According to a Business Day report, back then there were few local doctors serving in his community and little hope for young people from underprivileged backgrounds who aspired to a medical career. Today he is the acting clinical manager at Ganyesa District Hospital near Vryburg, and five of the eight doctors employed at the rural facility are Cuban-trained South Africans.

‘Obviously it was not an easy route, but rural areas now have permanent South African doctors who can speak the language of their patients. Some of us would never have had the opportunity to study medicine in South Africa,’ Kegakilwe says.

The report says South Africa began sending aspirant doctors to train in Cuba in 1997 under a deal that also saw Cuba send its doctors to work in South Africa’s rural areas. The South Africans joined students from all over the world taking advantage of the many medical schools created under Fidel Castro’s watch to provide personnel for his country’s free universal healthcare system.

South Africa recruited bright young people from underprivileged backgrounds and sent them to Cuba on bursaries that required them to work for an equal number of years in the state sector after they had qualified. But, the report says, the programme was contentious from the beginning, as the ANC-led government maintained close political ties to Castro, in recognition of his support for the organisation when it was in exile during apartheid.

Critics questioned the wisdom of training doctors in a country with a vastly different burden of disease, along with a completely different approach to healthcare that emphasised prevention rather than cure. The government’s defence was that South Africa needed more doctors, and local medical schools did not have the capacity to immediately expand their student intake.

The initiative was neither efficient nor cheap, the report says. Although the cost of living was lower in Cuba, the students took at least two years longer to qualify than their locally trained peers. They spent a year learning Spanish before they could begin their medical training and required more time to adjust to South African medical schools after their return. Although the students obtained a Cuban medical degree, they were required to pass South African final-year medical exams to graduate and register with the Health Professions Council of South Africa.

Many students found the adjustment to life in Cuba daunting, and their return to South Africa as difficult. ‘The South African students were more fluent with the terminology, the equipment was different, and we were thinking in Spanish. ‘But the worst part was the lecturers: they didn’t provide support and (some) would tell us to our faces, &quot;you are dumb … you will fail&quot;,’ Kegakilwe is quoted in the report as saying.

His cohort was ill-prepared for the trauma and infectious diseases affecting South African patients, particularly the horror of the HIV epidemic. ‘It was before the ARV (antiretroviral) roll-out and we were seeing patients with fullblown AIDS and its complications,’ he says. ‘But if you have the basics right, wherever you go, you can adapt,’ Kegakilwe says.

The report says now Cuban-trained students appear to be getting a better reception from medical students who trained locally than they did in the past. ‘When we came back, they taught us how to tackle questions and get used to the systems here. We integrate well, and the students don’t look down on you. But some lecturers say that we haven’t learnt enough skills,’ says Cedrick Thete, a Cuban-trained medical student from Bushbuckridge in Mpumalanga, who is completing his studies at the University of the Witwatersrand. ‘Yet the system in Cuba is better: they taught us how to work without technology, deal with prevention and study a community to identify risk factors for disease.’

Initially the number of students sent to Cuba each year was fairly small but, in 2012, Health Minister Aaron Motsoaledi announced an almost 10-fold increase in the size of the training programme. At that stage, South Africa’s eight medical schools were producing a mere 1 200 graduates a year – a figure that remained flat for more than a decade despite the growing population and the soaring HIV epidemic. The plan then was to increase the number of students going to Cuba to about 500 a year.

In the end, the report says, a far higher figure left for Cuba and about 720 Cuban-trained students are due to return to South Africa in July – the biggest cohort to enrol into the system at once. While they were studying, medical schools have steadily increased their enrolments and 1 800 doctors are expected to graduate this year, according to Martin Veller, chair of the South African Committee of Medical Deans.

Medical schools and provincial health departments now have to gear up to integrate an unusually large number of students at undergraduate level and provide the clinical training platform they need to get vital hands-on experience. ‘We are wrestling with how to adapt the curriculum,’ says Lionel Green-Thompson, assistant dean for teaching and learning in the Faculty of Health Sciences at Wits University, who recently visited Cuba with deputy health minister Joe Phaahla to assess the programme. ‘Their skill set is very similar to South African-trained students, but this needs to be supplemented with additional competencies to deal with the different burden of disease in South Africa, which includes a high level of trauma not seen in Cuba. The capacity of these students for primary healthcare is greater and we don’t want to erode that ethos,’ he is quoted in the report as saying. Wits is expecting to take about 150 students for their final round of training.

Discussions are under way with the Treasury to ensure that provinces have the requisite budgets to provide for the increased number of internship and community service posts that will be required after the Cuban-trained students and the enlarged cohort of locally qualified doctors graduate, says the health department’s chief director for human resources, Gavin Steel. Internships have historically been conducted at large hospitals. However, they may in the future also take place at smaller facilities, while community service for Cuban-trained doctors is likely to take place in a primary healthcare setting, Steel says.

Motsoaledi said recently that the Cuban doctor-training programme was so big it was a headache for both countries, and the National Health Council had decided it should be temporarily scaled back but Steel says in the report that it is likely that there will always be a place for Cuban medical training but the numbers are likely to diminish significantly as local training capacity grows.

‘The programme had two targets: increase the number of medical graduates and provide opportunities for kids from disadvantaged backgrounds. If you look at it from that perspective it has been a success,’ he says.
